# Ocular toxoplasmosis in Latin American and European patients: clinical characteristics, visual outcomes, and recurrence patterns

**DOI:** 10.1186/s12348-026-00615-9

**Published:** 2026-06-26

**Authors:** Arman Shamani-Aminian, Alfredo Adán, Víctor Llorenç

**Affiliations:** 1 University of Barcelona, Campus Hospital Clínic, Barcelona, Spain; 2 Institut Clínic d’Oftalmologia, Hospital Clínic de Barcelona, Barcelona, Spain; 3 Institut d’Investigacions Biomèdiques August Pi i Sunyer (IDIBAPS), Barcelona, Spain; 4 Ocular Inflammation and Infection Section, Institut Clínic d’Oftalmologia, Hospital Clínic de Barcelona, C/ Sabino de Arana 1 Maternity Hospital Building, 2nd Floor, Barcelona, 08028 Spain

**Keywords:** Ocular toxoplasmosis, Retinochoroiditis, Posterior uveitis, *Toxoplasma gondii*, Latin American patients, Atypical toxoplasmosis, Uveitis

## Abstract

**Background:**

Ocular toxoplasmosis is the most common cause of posterior uveitis worldwide and a major cause of visual impairment. Previous studies suggest that the disease may follow a more aggressive clinical course in patients from Latin America; however, direct comparative evidence between geographic populations remains limited. This study aimed to characterize the clinical, serological, and imaging features of a multiethnic cohort of patients with ocular toxoplasmosis and to assess differences according to geographic origin.

**Methods:**

A retrospective chart review was performed including 144 patients diagnosed with ocular toxoplasmosis at a tertiary referral center in Barcelona, Spain. Clinical characteristics, recurrence patterns, visual outcomes, and serological findings were analyzed. Outcomes were compared between Latin American (*n* = 73) and European (*n* = 71) patients.

**Results:**

Latin American origin (OR 8.21; *p* < 0.001) and older age at disease onset (OR 1.04; *p* = 0.009) were independently associated with atypical disease presentation. Latin American origin (β = +0.20 LogMAR; *p* = 0.02), congenital disease (β = +0.52 LogMAR; *p* < 0.001), and retinal zone I involvement (β = +0.57 LogMAR; *p* < 0.001) were independently associated with worse visual acuity outcomes. Latin American origin (OR 4.85; *p* = 0.002) and longer time since disease onset (OR 1.05; *p* = 0.04) were independently associated with multiple recurrences, whereas congenital disease (OR 0.03; *p* = 0.01) was associated with lower recurrence risk. Serum IgG levels were significantly higher in Latin American patients (*p* = 0.002), who also more frequently required systemic corticosteroids along with antiparasitic treatment.

**Conclusions:**

Patients of Latin American origin showed a higher prevalence of atypical disease, increased recurrence rates, and poorer visual outcomes compared with European patients. These findings support the hypothesis of a more severe disease phenotype in this population and highlight the importance of closer monitoring and individualized management strategies in patients at higher risk of severe ocular toxoplasmosis.

**Supplementary Information:**

The online version contains supplementary material available at https://doi.org/10.1186/s12348-026-00615-9.

## Introduction

Ocular toxoplasmosis (OT) is the most common cause of posterior uveitis worldwide and a major cause of visual impairment [1–4]. The disease results from infection with the intracellular protozoan *Toxoplasma gondii*, a ubiquitous parasite with a global distribution. Serological studies suggest that up to one-third of the world’s population has been exposed to the parasite, although most infections remain asymptomatic [5]. In individuals who develop ocular involvement, the typical manifestation consists of a focal necrotizing retinochoroiditis, frequently accompanied by vitritis and other signs of intraocular inflammation [3, 6–9].

Despite this characteristic presentation, the clinical spectrum of OT is heterogeneous. Atypical forms may occur, including large or multifocal retinal lesions, bilateral disease, punctate outer retinal lesions, and other unusual inflammatory patterns that may complicate the diagnosis [8, 10, 11]. In addition to clinical variability, the course of the disease is often characterized by recurrent inflammatory episodes that may lead to progressive retinal damage and visual loss [12].

Increasing evidence suggests that geographic origin may influence the clinical expression and severity of OT. Several studies have reported that patients from Latin America tend to develop more severe disease, including larger retinal lesions, higher recurrence rates, and poorer visual outcomes compared with patients from Europe or North America [13–16]. These differences have been partly attributed to the greater genetic diversity of *T. gondii* strains circulating in South America, where atypical and highly virulent parasite lineages are more prevalent [17, 18].

Comparative studies have also reported differences in clinical presentation and long-term visual outcomes between patients from different geographic backgrounds [14, 17, 18]. However, relatively few studies have evaluated Latin American and European patients within the same clinical setting. In addition, studies integrating detailed clinical characterization with imaging findings and serological data remain limited.

A better understanding of these differences may help identify epidemiological and clinical patterns associated with disease severity and recurrence. Such insights could contribute to improved risk stratification and management strategies in patients with OT.

We hypothesized that patients born in Latin America and evaluated in Spain are more likely to present with atypical and more severe manifestations of OT, as well as a higher risk of recurrent disease, compared with patients born in Europe. The aim of this study was therefore to compare the clinical, serological, and demographic characteristics of a multinational cohort of patients with OT of Latin American and European origin evaluated at a tertiary referral center in Barcelona using a retrospective chart review design.

## Methods

### Study design and setting

This study was designed as a retrospective observational chart review with both descriptive and analytical components. It was conducted at a single tertiary referral center, Hospital Clínic de Barcelona (Barcelona, Spain), and included patients diagnosed with OT between January 2015 and August 2025.

### Patient selection

Patients diagnosed with OT at our institution during the study period were eligible for inclusion regardless of age, sex, or ethnic background, provided that sufficient clinical information was available in the medical records and that the country or geographic area of birth was recorded.

The diagnosis of OT was established based on characteristic funduscopic findings consistent with toxoplasmic retinochoroiditis, the exclusion of alternative etiologies, and the presence of positive anti–*Toxoplasma gondii* IgG antibodies. Additional confirmatory tests, including polymerase chain reaction (PCR) analysis of aqueous or vitreous humor or calculation of the Goldmann–Witmer coefficient, were performed at the discretion of the treating clinician in diagnostically uncertain cases.

Patients initially diagnosed at other institutions but subsequently referred to our center for follow-up or emergency care were also eligible for inclusion.

Patients were excluded if medical records contained insufficient clinical information or conflicting or ambiguous data that precluded reliable evaluation. For outcome assessment, patients with less than 12 months of follow-up were excluded.

### Data collection and imaging assessment

All data were obtained from electronic medical records and available diagnostic imaging. Imaging modalities primarily included ultra-widefield (UWF) pseudocolor fundus photography and blue-light autofluorescence imaging (Optos^®^, Nikon, UK), as well as spectral-domain optical coherence tomography (SD-OCT; Cirrus^®^, Carl Zeiss Meditec, Germany) using the macular cube acquisition protocol.

Ultra-widefield fluorescein angiography (UWF-FA) and/or high-definition raster OCT scans were performed at the discretion of the treating clinician based on initial clinical findings.

No additional clinical visits, examinations, or diagnostic procedures were performed specifically for the purposes of this study.

### Study variables

The primary variable of interest was geographic origin (Latin American vs. European). The main clinical outcomes included disease typicity (typical vs. atypical presentation), baseline and final visual acuity, recurrence patterns, treatment modalities, treatment response, and ocular sequelae.

Atypical disease presentation was defined by the presence of one or more of the following features: extensive retinal lesions (≥ 3-disc diameters), multifocal lesions, bilateral involvement, panuveitis, or neuroretinitis, as previously described [6, 10, 19].

Baseline visual acuity was defined as the best corrected or pinhole visual acuity recorded at the first visit at our center, and final visual acuity as the best recorded value at the last available follow-up visit. Visual acuity was initially recorded using decimal Snellen charts and subsequently converted to logarithm of the minimum angle of resolution (LogMAR) units for statistical analysis.

Recurrence patterns were classified as non-recurrent disease, a single recurrence, or multiple recurrences during follow-up.

Treatment modalities included antiparasitic therapy, systemic and topical corticosteroids, prophylactic therapy, and intravitreal treatment (antiparasitic ± corticosteroid).

Secondary variables included sex, age, date of birth, serological profile (IgM positivity, IgG levels, and IgG avidity), retinal zone involvement according to the UCLA classification, presence and number of chorioretinal scars, previous ocular history, reason for consultation, immune status, and dates of the first and last clinical visits.

The following structural complications were evaluated as secondary outcomes: macular edema, choroidal neovascular membrane, and retinal detachment.

### Statistical analysis

Ocular characteristics and visual outcomes were analyzed per affected eye, whereas demographic and systemic variables were analyzed per patient. Descriptive statistics were used to summarize demographic and clinical characteristics. The distribution of continuous variables was assessed using the Shapiro–Wilk test.

As several variables were not normally distributed, comparisons between groups were performed using the Mann–Whitney U test for continuous variables and the chi-square test or Fisher’s exact test for categorical variables, as appropriate.

For the analysis of IgG levels, values exceeding the upper limit of quantification (> 700 IU/mL) were truncated at 700 IU/mL.

To identify independent predictors of atypical disease presentation, visual acuity outcomes, and recurrence patterns, multivariable regression models were constructed. Multiple linear regression was used to evaluate baseline and final visual acuity (LogMAR). Binary logistic regression was used to identify predictors of atypical versus typical presentation. Recurrence patterns were analyzed using multinomial logistic regression.

For analyses of visual acuity, both eyes were included as independent observations in patients with bilateral disease. No adjustment for intra-patient correlation was performed, as no significant correlation was observed between eyes in bilateral cases at baseline or final visual acuity. The potential impact of this approach was considered minimal due to the low proportion of bilateral cases. In contrast, analyses of recurrence and disease typicity were conducted at the patient level.

The covariates included in each multivariable model are specified in the Results section. Statistical significance was defined as a two-sided p-value < 0.05. Results were reported as odds ratios (OR) or β coefficients with 95% confidence intervals (CI). Analyses were conducted using complete-case data. Statistical analyses were performed using SPSS software (version 27; IBM Corp., Armonk, NY, USA).

### Ethical considerations

The study adhered to the tenets of the Declaration of Helsinki and was approved by the Institutional Review Board of Hospital Clínic de Barcelona. Given the retrospective nature of the study and the use of anonymized clinical data, the requirement for informed consent was waived.

## Results

### Patient characteristics

Out of 158 patients diagnosed with OT during the study period, 14 were born outside Latin America or Europe and were excluded. The final study cohort included 144 patients, of whom 84 were female (58%) and 60 were male (42%). Seventy-one patients (49%) were born in Europe and 73 (51%) in Latin American countries.

**Fig. 1:**
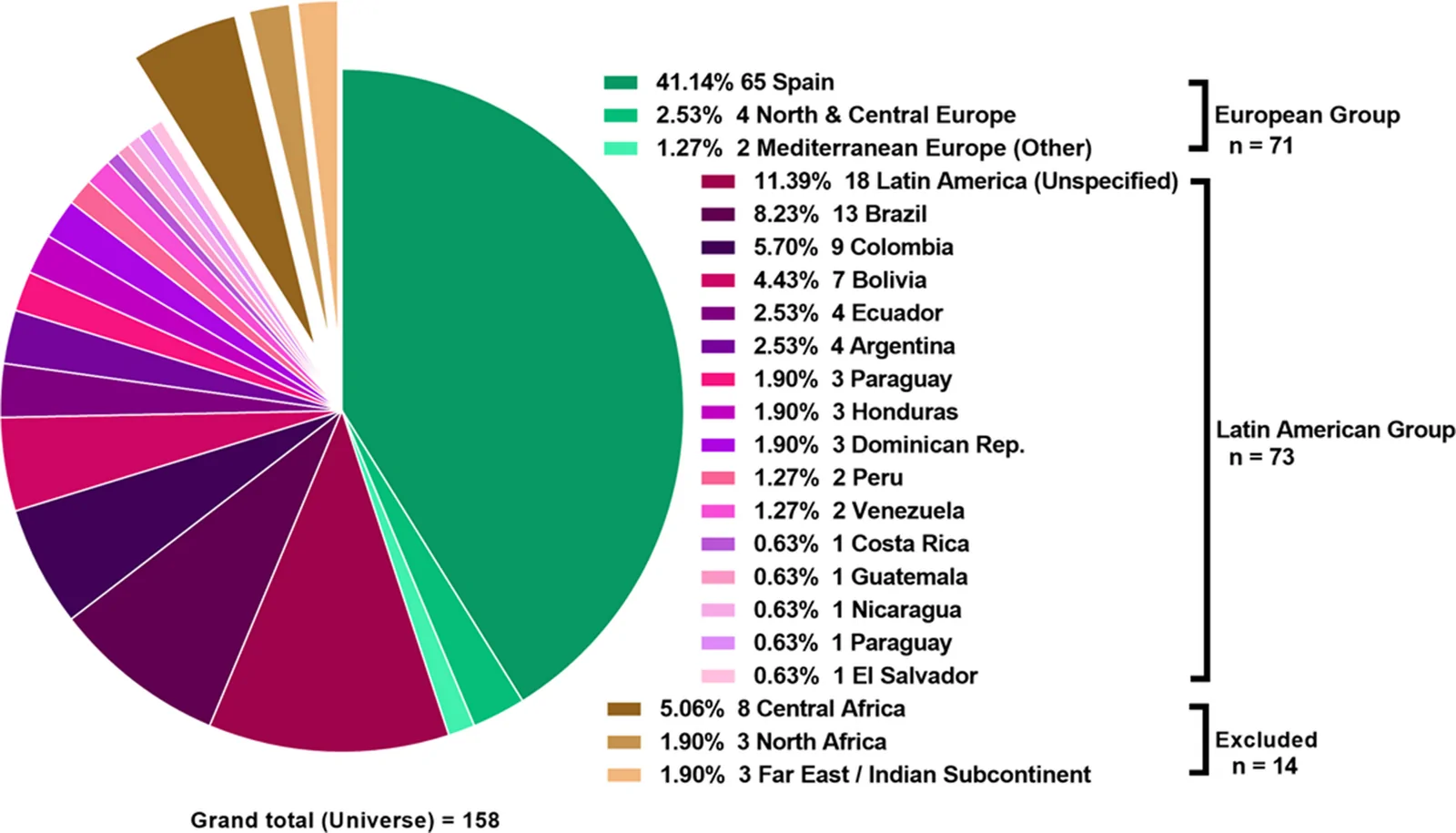
Geographic origin of the study population. Distribution of patients screened for ocular toxoplasmosis according to country of birth. Patients were categorized into European (*n* = 71) and Latin American (*n* = 73) groups for comparative analysis. Individuals born in African countries (*n* = 11) and Southeast Asia (*n* = 3) were excluded

The most frequent Latin American countries of origin were Brazil (*n* = 13; 9%) and Colombia (*n* = 9; 6%). In 18 Latin American patients, the specific country of birth was not recorded (Fig. 1). Thirteen patients were immunosuppressed at the time of their first evaluation.

The median age at baseline was 45 years (IQR 35–57), and the median age at disease onset was 30 years (IQR 21–40). European-born patients were older than Latin American patients (53 [IQR 36.5–66.5] vs. 41 [IQR 35–48] years). The median age at disease onset was 30.5 years (IQR 18.3–53.0) in European-born patients and 29 years (IQR 23.0–36.0) in Latin American patients.

Baseline demographic characteristics are summarized in Table 1, and ocular findings are presented in Table 2. Additional ocular characteristics are provided in Supplementary Table S1.

**Table 1:** Baseline demographic characteristics of the study population according to geographic origin

Characteristic	All patients (*n* = 144)	Latin American (*n* = 73)	European (*n* = 71)	*p*-value
**Age at baseline**, median (IQR), years	45.0 (35.0–57.0)	41.0 (35.0–48.0)	53.0 (36.5–66.5)	< 0.001
**Age at disease onset**, median (IQR), years	30.0 (21.0–40.0)	29.0 (23.0–36.0)	30.5 (18.3–53.0)	0.06
**Male sex**, n (%)	60 (41.7)	29 (39.7)	31 (43.7)	0.63
**Female sex**, n (%)	84 (58.3)	44 (60.3)	40 (56.3)	0.63
**Immunosuppression**, n (%)	13 (9.0)	9 (12.3)	4 (6.2)	0.24
**HIV infection**, n (%)	6 (4.2)	5 (6.8)	1 (1.5)	0.21

Abbreviations: IQR, interquartile range

**Table 2:** Clinical characteristics and disease presentation according to geographic origin

Characteristic		All patients (*n* = 144)		Latin American (*n* = 73)		European (*n* = 71)			*p*-value
Laterality									
**Unilateral** disease, n (%)		121 (84.0)		64 (87.7)		57 (80.3)			0.23
– **Left eye**, n (%)		55 (45.5)		29 (45.3)		26 (45.6)			0.97
– **Right eye**, n (%)		66 (54.5)		35 (54.7)		31 (54.4)			0.97
**Bilateral** disease, n (%)		23 (16.0)		9 (12.3)		14 (19.7)			0.23
**Disease presentation**									
**Typical** presentation, n (%)		49 (34.0)		13 (17.8)		36 (50.7)			< 0.001
**Atypical** presentation, n (%)		91 (63.2)		57 (78.1)		34 (47.9)			< 0.001
Unknown¹, n (%)		4 (2.8)		3 (4.1)		1 (1.4)			0.62
**Atypical features**									
**Extensive retinal lesion**, n (%)		62 (43.1)		39 (53.4)		23 (32.3)			0.01
**Multifocal disease**, n (%)		33 (22.9)		19 (26.0)		14 (19.7)			0.37
**Bilateral involvement**, n (%)		23 (16.0)		9 (12.3)		14 (19.7)			0.23
**Panuveitis**, n (%)		23 (16.0)		13 (17.8)		10 (14.1)			0.54
**Neuroretinitis**, n (%)		2 (1.3)		1 (1.4)		1 (1.4)			1.00

Note: Atypical presentation was defined as the presence of one or more of the following features: extensive retinal lesions (≥ 3 disc diameters), multifocal lesions, bilateral involvement, panuveitis, or neuroretinitis. Patients could present with more than one atypical feature

¹Three Latin American patients and one European patient could not be classified due to insufficient clinical or imaging data

### Clinical presentation according to geographic origin

Latin American patients more frequently presented with symptomatic disease at the first visit. Blurred vision (23%), floaters (22%), and decreased vision (15%) were the most commonly reported symptoms. In contrast, European-born patients more often attended asymptomatic follow-up visits (24%).

Latin American patients showed higher rates of active disease at presentation compared with European-born patients (72% vs. 52%, *p* = 0.007).

Based on clinical history and serological results at the first visit, patients were categorized as primary infection, first reactivation, recurrence, congenital infection, or follow-up. Among Latin American patients, 55% were classified as first reactivation and 29% as recurrence, compared with 48% and 17%, respectively, among European-born patients (*p* = 0.41 for first reactivation; *p* = 0.09 for recurrence). Detailed distributions are provided in Supplementary Table S2.

Atypical manifestations were significantly more frequent among Latin American patients (78% vs. 48%, *p* < 0.001). The most common atypical features in Latin Americans, included extensive retinal lesions (53.4%), multifocal disease (26%), and panuveitis (17.8%) (Table 2).

**Fig. 2:**
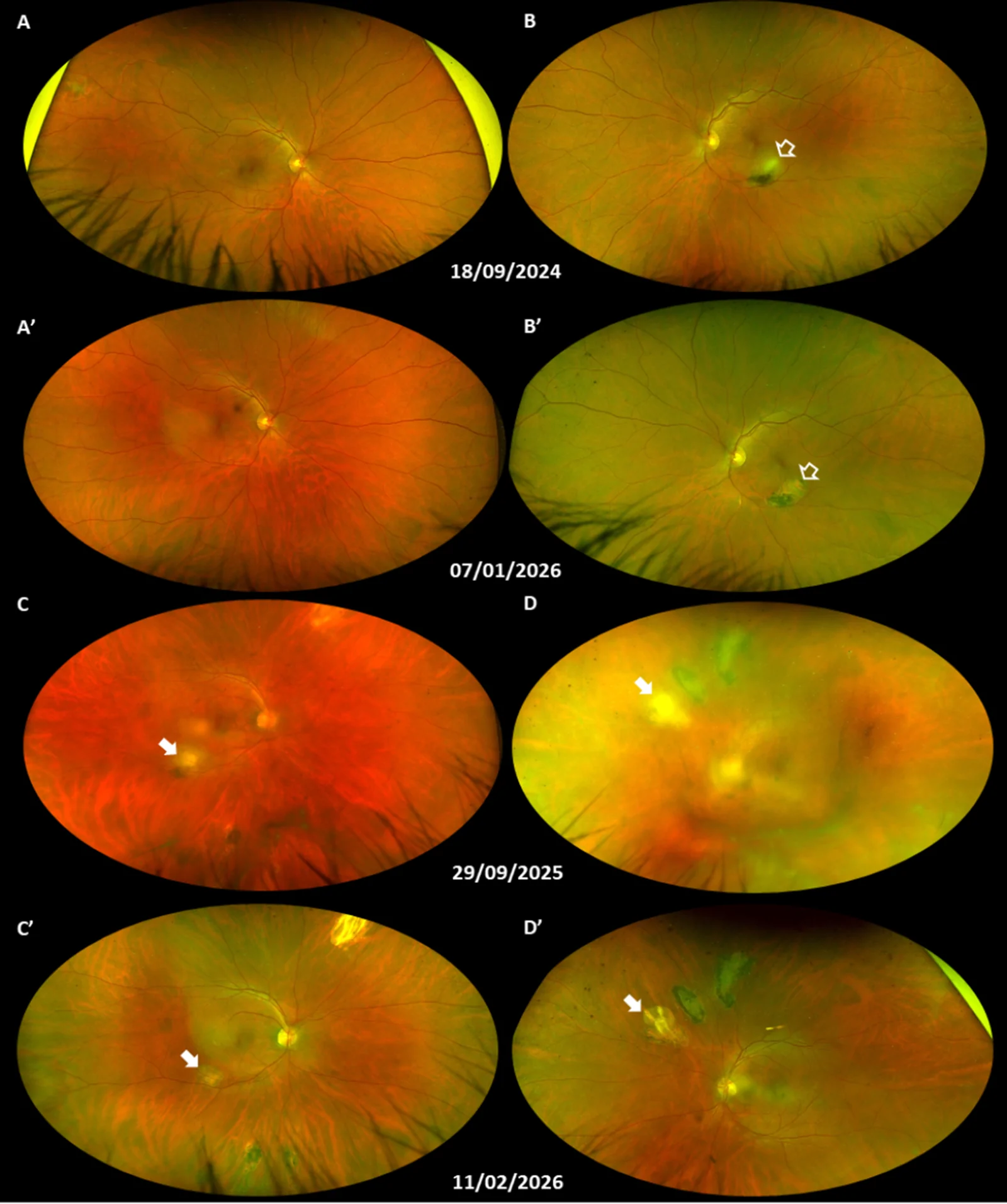
Typical and atypical ocular toxoplasmosis. Ultra-widefield (UWF) pseudocolor fundus images obtained using Optos^®^ (Nikon, UK). (**A, B**) Typical ocular toxoplasmosis in a Spanish-born patient. Panel A shows a normal fundus in the right eye (OD), whereas panel B shows an active retinochoroidal lesion in the left eye (OS), appearing as a small yellowish-white lesion adjacent to a hyperpigmented scar with mild overlying vitreous haze. The lesion resolved after 8 weeks of oral cotrimoxazole treatment, leaving an atrophic chorioretinal scar (**A**′, **B**′; empty arrows). (**C, D**) Atypical ocular toxoplasmosis in a Colombian-born patient. Bilateral active yellowish retinochoroidal lesions associated with marked vitreous haze are observed, particularly in the left eye. Follow-up images obtained after 16 weeks of combined systemic cotrimoxazole and prednisone therapy, together with a single intravitreal injection of clindamycin and dexamethasone in the left eye, show multiple extensive chorioretinal scars in both eyes (**C**′, **D**′; filled arrows)

In multivariable logistic regression analysis—including geographic origin, age at disease onset, sex, immunosuppression status, retinal zone involvement, and lesion activity—Latin American origin (OR 8.21; 95% CI 3.30–20.4; *p* < 0.001) and older age at disease onset (OR 1.04; 95% CI 1.01–1.07; *p* = 0.009) were independently associated with atypical disease (Fig. 2).

### Visual acuity

Median baseline visual acuity was worse in Latin American patients (0.40 LogMAR [IQR 0.10–1.00]) compared with European-born patients (0.22 LogMAR [IQR 0.05–0.80]) (*p* = 0.03).

In multivariable linear regression analysis—including geographic origin, age at disease onset, sex, congenital infection, and immunosuppression status—Latin American origin (β = +0.20 LogMAR; 95% CI 0.03–0.36; *p* = 0.02) and congenital disease (β = +0.37 LogMAR; 95% CI 0.13–0.61; *p* = 0.002) were independently associated with worse baseline visual acuity.

### Serological findings

Serum IgG levels were significantly higher in Latin American patients compared with European-born patients (*p* = 0.002). No significant differences in IgG avidity were observed between groups. Detailed serological data are provided in Supplementary Table S3.

### Treatment

Latin American patients more frequently required systemic corticosteroids during antiparasitic therapy compared with European-born patients (70% vs. 46%, *p* = 0.005). Intravitreal therapy was rarely required in both groups. Detailed treatment regimens are presented in Supplementary Table S4.

### Recurrences and visual outcomes

For recurrence analysis, patients with less than 12 months of follow-up were excluded. A total of 55 Latin American and 61 European patients were included in the final analysis of recurrences. Median follow-up was 73 months (IQR 37–150) in Latin American patients and 92 months (IQR 40–170) in European-born patients.

Multiple recurrences occurred more frequently among Latin American patients (38% vs. 19%, *p* = 0.03).

In multinomial logistic regression analysis—including geographic origin, time since disease onset, sex, congenital infection, and immunosuppression status—multiple recurrences were independently associated with Latin American origin (OR 4.85; 95% CI 1.80–13.1; *p* = 0.002) and longer disease duration (OR 1.05; 95% CI 1.002-1.10; *p* = 0.04). Congenital disease (OR 0.03; 95% CI 0.002–0.48; *p* = 0.01) was associated with lower odds of multiple recurrences.

Median final visual acuity was 0.15 LogMAR (IQR 0.00–0.70) in Latin American patients and 0.06 LogMAR (IQR 0.00–0.70) in European-born patients (*p* = 0.40).

In multivariable linear regression analysis—including geographic origin, age at disease onset, sex, congenital infection, retinal zone involvement, and immunosuppression status—final visual acuity was independently associated with zone I involvement (β = +0.57 LogMAR; 95% CI 0.39–0.75; *p* < 0.001), and Latin American origin (β = +0.20 LogMAR; 95% CI 0.03–0.36; *p* = 0.02).

Detailed recurrence data are presented in Supplementary Table S5.

### Structural complications

Macular edema occurred in 7 Latin American eyes and 4 European eyes (*p* = 0.53). Retinal detachment was observed in 2 Latin American eyes and 1 European eye (*p* = 0.29). One case of choroidal neovascular membrane was reported in each group (*p* = 1.00).

## Discussion

To our knowledge, this is one of the largest comparative cohorts of Latin American and European patients with OT evaluated within the same European tertiary referral center.

This study provides a comparative analysis of the clinical, imaging, and serological characteristics of OT according to geographic origin in a cohort managed at a European tertiary referral center. Our findings indicate that patients born in Latin America tend to present with more severe manifestations of OT compared with European-born patients. Specifically, Latin American origin was associated with a higher frequency of atypical disease presentation, an increased risk of recurrent episodes, and worse baseline and final visual acuity. These associations remained significant or reached significance after adjustment for relevant clinical covariates in multivariable analyses, including age at disease onset, sex, retinal zone involvement, congenital infection, and immunosuppression status.

Latin American patients more frequently presented with active lesions at the time of consultation and required more intensive treatment, including antiparasitic therapy combined with systemic corticosteroids. Serum IgG levels were also significantly higher in this group. This finding should be interpreted with caution, as it may reflect the greater clinical severity observed in these patients rather than a direct causal relationship. Higher IgG titers may indicate a greater cumulative antigenic burden, potentially related to early-life exposure in highly endemic regions and repeated environmental exposure to *Toxoplasma gondii*. In addition, the distinct genotypic landscape of *T. gondii* in Latin America—characterized by recombinant, non-Type II strains—may contribute to both increased disease severity and a more pronounced humoral immune response [20, 21]. Consequently, elevated IgG levels in this population may represent a surrogate marker of more aggressive infection, although this hypothesis requires further investigation. To our knowledge, few studies have directly compared quantitative IgG levels between Latin American and European patients.

In contrast, the overall distribution of structural sequelae was similar between groups. Although macular scarring appeared more frequent among European patients, this difference was likely influenced by the higher proportion of congenital cases in this subgroup, which are strongly associated with macular involvement [22]. When congenital cases were excluded, the prevalence of macular scarring became comparable between groups.

Among additional predictors, Latin American origin, and retinal zone I involvement were independently associated with worse visual outcomes and a higher probability of multiple recurrences [23]. These findings are consistent with previous reports describing increased severity of OT in Latin American populations [14, 17, 18]. For example, Gilbert et al. reported more severe ocular involvement among congenitally infected children in Brazil compared with European cohorts [17]. Similarly, a recent systematic review and meta-analysis found higher recurrence rates and a greater prevalence of visual impairment related to OT in South and Central America compared with Europe [24]. However, these studies also highlighted methodological limitations, including heterogeneity in study design and variability in the reporting of visual acuity outcomes.

Several mechanisms may explain the increased severity of OT observed in Latin American patients. One key factor is the high genetic diversity of *Toxoplasma gondii* strains circulating in Central and South America [25]. These strains often exhibit recombinant genotypes associated with increased virulence compared with the clonal lineages typically found in Europe and North America. For instance, Khan et al. demonstrated that Brazilian strains are genetically distinct from dominant European lineages [25]. Similarly, Shobab et al. reported that parasite serotype may influence disease severity [26]. De la Torre et al. further showed that Colombian patients with active OT harbored heterogeneous atypical strains and exhibited a distinct intraocular cytokine profile, characterized by lower IFN-γ and IL-17 levels and higher IL-6 and IL-13 levels, suggesting an immune response that may contribute to more severe disease [18].

The present study offers several strengths. First, it compares Latin American and European-born patients within the same tertiary referral center, thereby minimizing confounding related to differences in healthcare systems, diagnostic approaches, and treatment protocols. Second, it includes a relatively large European cohort with a substantial proportion of Latin American patients, allowing a balanced comparison between populations. Third, the integration of clinical, imaging, and serological data provides a comprehensive characterization of disease presentation and outcomes.

Several limitations should be acknowledged. As a single-center study conducted in a tertiary referral institution, the cohort may overrepresent patients with more severe or atypical disease, potentially limiting generalizability. In addition, in the multivariable analysis of visual acuity, both eyes were included as independent observations in patients with bilateral disease, which may introduce intra-patient correlation bias. However, the impact of this approach is likely limited given the low proportion of bilateral cases and the absence of significant correlation between eyes.

Furthermore, country of birth should not be interpreted as a direct determinant of OT manifestations, but rather as a proxy for several biological, social, and environmental factors. In addition to geographic differences in Toxoplasma gondii strain diversity and virulence, factors such as environmental exposure, migration history, socioeconomic conditions, healthcare access, healthcare-seeking behaviour, referral pathways, ophthalmologic care in the country of origin, and follow-up patterns may have contributed to the observed differences between Latin American and European patients.

Finally, although all eligible patients were included to reduce selection bias, some subgroups—such as immunocompromised patients—remained relatively small.

In conclusion, patients born in Latin America and evaluated at a European tertiary referral center showed a higher prevalence of atypical disease presentation, increased recurrence rates, and poorer visual outcomes compared with European-born patients with OT. These findings support the hypothesis that geographic background may influence disease expression and prognosis. Recognition of these differences may help guide clinical decision-making, including closer monitoring and individualized management strategies for patients at higher risk of severe disease.

Future multicenter prospective studies integrating parasite genotyping, host immune profiling, and environmental exposure data are needed to further elucidate the determinants of severe OT and to improve prevention and management strategies.

## Supplementary Information

Below is the link to the electronic supplementary material.

Supplementary Material 1

## Data Availability

The datasets generated and/or analyzed during the current study are not publicly available due to institutional restrictions regarding clinical data but are available from the corresponding author on reasonable request.
