# Supplementary material for: Ocular toxoplasmosis in Latin American and European patients: clinical characteristics, visual outcomes, and recurrence patterns
Source: J Ophthalmic Inflamm Infect. 2026 Jun 26;16:56. doi: 10.1186/s12348-026-00615-9 (PMC13562453; doi:10.1186/s12348-026-00615-9)
Supplement: Supplementary file 1 [file 12348_2026_615_MOESM1_ESM.docx]

**Ocular toxoplasmosis in Latin American and European patients: clinical characteristics, visual outcomes, and recurrence patterns**

**SUPPLEMENTARY TABLES**

**Supplementary Table 1. Ocular characteristics and visual outcomes according to geographic origin (analysis per affected eye)**

| **Characteristic** | **All eyes**  **(n = 167)** | **Latin American**  **(n = 82)** | **European**  **(n = 85)** | **p-value** |
| --- | --- | --- | --- | --- |
| **Initial visual acuity** (**LogMAR**), median (IQR) | 0.30 (0.05–0.80) | 0.40 (0.10–1.00) | 0.22 (0.05–0.80) | 0.03 |
| ≥ 0.5 Snellen, n (%) | 73 (43.7) | 29 (35.4) | 44 (51.8) | 0.03 |
| 0.4–0.5 Snellen, n (%) | 24 (14.4) | 15 (18.3) | 9 (10.6) | 0.16 |
| 0.1–0.3 Snellen, n (%) | 30 (18.0) | 14 (17.1) | 16 (18.8) | 0.77 |
| ≤ 0.1 Snellen, n (%) | 40 (24.0) | 24 (29.3) | 16 (18.8) | 0.11 |
| **Active lesions**, n (%) | 103 (61.7) | 59 (72.0) | 44 (51.8) | 0.01 |
| **Retinal zone affected¹** |  |  |  |  |
| Zone I, n (%) | 53 (31.7) | 25 (30.5) | 28 (32.9) | 0.73 |
| Zone II, n (%) | 103 (61.7) | 54 (65.9) | 49 (57.6) | 0.28 |
| Zone III, n (%) | 28 (16.8) | 14 (17.1) | 14 (16.5) | 0.92 |
| Unknown/NA, n (%) | 16 (9.6) | 8 (9.8) | 8 (9.4) | 0.94 |
| **Number of chorioretinal scars** |  |  |  |  |
| None, n (%) | 25 (15.0) | 12 (14.6) | 14 (16.5) | 0.74 |
| One, n (%) | 74 (44.3) | 40 (48.8) | 34 (40.0) | 0.25 |
| Two, n (%) | 19 (11.4) | 6 (7.3) | 13 (15.3) | 0.10 |
| Three or more, n (%) | 24 (14.4) | 11 (13.4) | 13 (15.3) | 0.73 |
| Unknown, n (%) | 24 (14.4) | 13 (15.9) | 11 (12.9) | 0.59 |
| **Final visual acuity** (**LogMAR**), median (IQR) | 0.10 (0.00–0.70) | 0.15 (0.00–0.70) | 0.06 (0.00–0.70) | 0.40 |
| ≥ 0.5 Snellen, n (%) | 94 (56.3) | 46 (56.1) | 48 (56.5) | 0.96 |
| 0.4–0.5 Snellen, n (%) | 15 (9.0) | 8 (9.8) | 7 (8.2) | 0.73 |
| 0.1–0.3 Snellen, n (%) | 25 (15.0) | 11 (13.4) | 14 (16.5) | 0.58 |
| ≤ 0.1 Snellen, n (%) | 27 (16.2) | 16 (19.5) | 11 (12.9) | 0.25 |
| Unknown/NA, n (%) | 6 (3.6) | 1 (1.2) | 5 (5.9) | 0.21 |

**Notes:** Analyses were performed per affected eye. A total of 167 eyes from 144 patients were included.
¹Retinal zones defined according to the UCLA classification.

**Supplementary Table 2. Reason for consultation and symptoms according to geographic origin**

| **Characteristic** | **All patients**  **(n = 144)** | **Latin American**  **(n = 73)** | **European**  **(n = 71)** | **p-value** |
| --- | --- | --- | --- | --- |
| **Reason for consultation / symptoms** |  |  |  |  |
| Loss of vision, n (%) | 37 (25.7) | 19 (26.0) | 18 (25.4) | 1.00 |
| Floaters, n (%) | 49 (34.0) | 28 (38.4) | 21 (29.6) | 0.27 |
| Blurred vision, n (%) | 42 (29.2) | 29 (39.7) | 13 (18.3) | 0.01 |
| Photopsia/photophobia, n (%) | 19 (13.2) | 13 (17.8) | 6 (8.5) | 0.10 |
| Red eye, n (%) | 10 (6.9) | 7 (9.6) | 3 (4.2) | 0.33 |
| Ocular pain, n (%) | 19 (13.2) | 15 (20.5) | 4 (5.6) | 0.01 |
| Scotoma, n (%) | 11 (7.6) | 5 (6.9) | 6 (8.5) | 0.76 |
| Metamorphopsia, n (%) | 3 (2.1) | 2 (2.7) | 1 (1.4) | 1.00 |
| Headache, n (%) | 1 (0.7) | 1 (1.4) | 0 (0.0) | 1.00 |
| Foreign body sensation, n (%) | 1 (0.7) | 1 (1.4) | 0 (0.0) | 1.00 |
| Follow-up/asymptomatic, n (%) | 28 (19.4) | 7 (9.6) | 21 (29.6) | <0.001 |
| **Type of infection** |  |  |  |  |
| Primary infection, n (%) | 7 (4.9) | 2 (2.7) | 5 (7.0) | 0.27 |
| First reactivation, n (%) | 72 (50.0) | 40 (54.8) | 34 (47.9) | 0.41 |
| Recurrent disease, n (%) | 30 (20.8) | 21 (28.8) | 12 (16.9) | 0.09 |
| Congenital infection, n (%) | 15 (10.4) | 5 (6.9) | 10 (14.1) | 0.18 |
| Follow-up, n (%) | 14 (9.7) | 5 (6.9) | 9 (12.7) | 0.27 |
| Unknown¹, n (%) | 1 (0.7) | 0 (0.0) | 1 (1.4) | 0.49 |

**Note:** Patients could report more than one symptom.
¹One European patient could not be classified due to insufficient clinical information.

**Supplementary Table 3. Serological findings according to geographic origin**

| **Characteristic** | **All patients**  **(n = 144)** | **Latin American**  **(n = 73)** | **European**  **(n = 71)** | **p-value** |
| --- | --- | --- | --- | --- |
| Positive IgM, n (%) | 9 (6.3) | 3 (4.1) | 6 (8.5) | 0.32 |
| IgG level, median (IQR), IU/mL | 252.2 (57.9–377.5) | 299.8 (89.0–483.3) | 168.9 (41.4–210.2) | 0.002 |
| IgG avidity index, median (IQR) | 0.55 (0.48–0.63) | 0.58 (0.53–0.65) | 0.50 (0.44–0.63) | 0.07 |

**Notes:** IgM data were unavailable in 24 patients. IgG levels were unavailable in 24 patients. IgG avidity was assessed in 100 patients. Values >700 IU/mL were truncated to 700 IU/mL.

**Supplementary Table 4. Treatment modalities according to geographic origin**

| **Characteristic** | **All patients**  **(n = 144)** | **Latin American**  **(n = 73)** | **European**  **(n = 71)** | **p-value** |
| --- | --- | --- | --- | --- |
| Antiparasitic treatment, n (%) | 104 (72.2) | 60 (82.2) | 44 (62.0) | 0.007 |
| Systemic corticosteroid therapy, n (%) | 84 (58.3) | 51 (69.9) | 33 (46.5) | 0.004 |
| Prophylactic antiparasitic therapy, n (%) | 14 (9.7) | 5 (6.9) | 9 (12.7) | 0.27 |
| Intravitreal treatment, n (%) | 7 (4.9) | 4 (5.5) | 3 (4.2) | 1.00 |

**Note:** Antiparasitic therapy included trimethoprim–sulfamethoxazole or other standard regimens. Intravitreal treatment included clindamycin with or without dexamethasone.

**Supplementary Table 5. Recurrence patterns according to geographic origin**

| **Characteristic** | **All patients**  **(n = 144)** | **Latin American**  **(n = 73)** | **European**  **(n = 71)** | **p-value** |
| --- | --- | --- | --- | --- |
| No recurrence, n (%) | 49 (34.0) | 17 (23.3) | 32 (45.1) | 0.005 |
| One recurrence, n (%) | 26 (18.1) | 10 (13.7) | 16 (22.5) | 0.17 |
| Two recurrences, n (%) | 14 (9.7) | 11 (15.1) | 3 (4.2) | 0.046 |
| Three or more recurrences, n (%) | 27 (18.8) | 17 (23.3) | 10 (14.1) | 0.16 |
| Excluded*, n (%) | 28 (19.4) | 18 (24.7) | 10 (14.1) | 0.11 |

Note: *All patients were excluded due to < 12 months of follow up.
